# Melatonin as a Pleiotropic Modulator of Mitochondrial Function and Cellular Signaling in Ischemic Brain Injury

**DOI:** 10.3390/cells15121084

**Published:** 2026-06-15

**Authors:** Georgina Ortiz-Martínez, Luis Fernando Ortega-Varela, María Esther Olvera-Cortés, Miguel Russi-Hernández, Socorro Azarell Anzures-Gutiérrez, Santos Ramírez-Medina, Laura María Rosas-Ponce, José Miguel Cervantes-Alfaro

**Affiliations:** 1 Medical Coordination, Hospital General de Zona No. 83, OOAD Michoacán, Instituto Mexicano del Seguro Social (IMSS), Morelia 58260, Mexico; 2 Laboratorio de Analgesia y Mecanismos del Dolor, Facultad de Atención Integral en Ciencias de la salud y Asistencia Social, Universidad Michoacana de San Nicolás de Hidalgo (UMSNH), Morelia 58030, Mexico; 3 Laboratorio de Neurofisiología Clínica y Experimental, Centro de Investigación Biomédica de Michoacán, OOAD Michoacán, Instituto Mexicano del Seguro Social (IMSS), Morelia 58260, Mexico; 4 Emergency Department, Unidad Médica de Alta Especialidad, Hospital General, Centro Médico Nacional La Raza, OOAD México Norte, Instituto Mexicano del Seguro Social (IMSS), Mexico City 02990, Mexico; 5 Department of Pediatrics, Unidad Médica de Alta Especialidad No. 48, OOAD Guanajuato, Instituto Mexicano del Seguro Social (IMSS), León 37320, Mexico; azarell77@hotmail.com; 6 Emergency Department, Hospital General Regional No. 1, OOAD Michoacán, Instituto Mexicano del Seguro Social (IMSS), Charo 58200, Mexico; 7 Independent Researcher, Morelia 58000, Mexico

**Keywords:** stroke, melatonin, neuroprotection, oxidative stress, apoptosis, inflammation

## Abstract

**Highlights:**

What are the main findings?

- Melatonin exerts pleiotropic neuroprotective properties, including antioxidant, anti-inflammatory, mitochondrial regulation, and immunomodulatory effects.
- Experimental evidence consistently shows reduced infarct volume, improved neurological outcomes, and modulation of apoptosis, oxidative stress, and neuroinflammation.

What are the implications of the main findings?

- The temporal dynamics of ischemic stroke suggest that melatonin may act across multiple phases, from acute injury to subacute neurorepair and plasticity.
- Melatonin represents a promising adjunctive therapeutic candidate, further clinical studies are required to establish its impact on functional outcomes.

**Abstract:**

Acute ischemic stroke is one of the leading causes of mortality and disability globally, characterized by a complex and temporally structured cascade of cellular and molecular events. Although reperfusion therapies have improved outcomes, their narrow therapeutic window and limited availability leave many patients without effective treatment, highlighting the need for effective neuroprotective strategies capable of targeting multiple interconnected pathways. Melatonin has been proposed as a potential adjunctive neuroprotective agent based on its pleiotropic properties, modulating cellular signaling networks including antioxidant, anti-inflammatory, mitochondrial stabilizing, and BBB-preserving effects. Melatonin regulates key signaling pathways, thereby coordinating cellular responses to injury in multiple stages of ischemic pathophysiology, positioning it as a potential adjunctive therapy. Preclinical studies consistently demonstrate reductions in infarct volume, preservation of neuronal architecture, and improvements in neurological outcomes. However, clinical evidence remains limited to a small number of clinical trials, which suggest safety and possible early neurological benefit but are underpowered to determine long-term efficacy. Importantly, translational gaps persist regarding optimal dosing, duration of administration, and alignment with the temporal dynamics of post-ischemic injury. This review integrates current knowledge on the cellular and molecular mechanisms underlying the potential neuroprotective actions and its role as a pleiotropic modulator of ischemic injury.

## 1. Introduction

Ischemic stroke ranks among the top ten causes of morbidity, mortality, and disability worldwide [1]. Current treatment strategies are primarily aimed at recanalizing the occluded vessel, either through intravenous thrombolysis within the 4.5 h therapeutic window or mechanical thrombectomy for a subset of selected patients, up to 24 h after symptom onset [2].

However, a major challenge in managing ischemic stroke is that a significant proportion of patients do not arrive at emergency departments within the thrombolysis therapeutic window. This delay is frequently related to geographical barriers, delayed symptom recognition, inadequate hospital infrastructure, or lack of medical supplies [1]. Additionally, mechanical thrombectomy is not available in all hospitals [2]. These conditions highlight the urgent need to develop adjuvant or alternative therapies for use in different phases of the ischemic event.

Current treatment strategies focus on restoring cerebral blood flow through reperfusion therapies; they do not directly target the underlying pathophysiological cascade of ischemic stroke [2]. Multiple neuroprotective agents have been developed; however, no consistent clinical benefit has been demonstrated. These include glutamate antagonists (selfotel, aptiganel, eliprodil, and magnesium sulfate), antioxidants (*N*-acetylcysteine, tirilazad, citicoline, and vitamins C and E), anti-inflammatory agents (minocycline, fingolimod, natalizumab, and interleukin-1 receptor antagonists), apoptosis modulators (caspase inhibitors and erythropoietin), and pleiotropic neuroprotective agents such as cerebrolysin, ginsenoside Rg1, and statins. Although many of these agents showed promising results in preclinical models, their translation into effective clinical therapies has been limited, underscoring the complexity of ischemic brain injury and the challenges of neuroprotection in human stroke [3,4].

Despite extensive research efforts, effective neuroprotective therapies capable of mitigating secondary brain injury have not yet been successfully translated into routine clinical practice. The pathophysiology of ischemic stroke extends beyond the initial vascular occlusion and involves a complex cascade of oxidative stress, neuroinflammation, mitochondrial dysfunction, apoptotic signaling, and BBB disruption. These processes evolve over hours to days, suggesting a broader biological window for therapeutic intervention than currently utilized [5,6].

Melatonin, an endogenous indoleamine traditionally associated with circadian regulation, has emerged as a pleiotropic regulator of cellular homeostasis. Melatonin modulates interconnected signaling networks and targets mitochondrial dysfunction across the temporal evolution of ischemic injury [7].

Preclinical studies have shown that melatonin can reduce neuronal damage in animal models of cerebral ischemia, making it a potential neuroprotective compound to complement standard treatment [8,9,10]. Melatonin fulfills several criteria desirable in a neuroprotective agent for ischemic stroke: it has a favorable safety profile, can cross the BBB, demonstrates a high safety margin, is compatible with standard care, and is available in both oral and intravenous formulations [11,12].

This review examines the cellular and molecular mechanisms underlying melatonin’s neuroprotective effects and evaluates current preclinical and clinical evidence, highlighting key translational gaps and future directions for its investigation in acute ischemic stroke.

## 2. Methods

This study is a narrative review of the literature that prioritizes mechanistic, translational, and clinical evidence to provide an integrative perspective on the role of melatonin across the temporal evolution of ischemic stroke. Relevant articles were identified through searches in electronic databases including PubMed, Scopus, and Web of Science. Keywords included: melatonin, pharmacokinetics, ischemic stroke, neuroprotection, oxidative stress, neuroinflammation, mitochondrial dysfunction, blood–brain barrier, apoptosis, reperfusion injury, cellular signaling pathways, and melatonin receptors.

Multiple combinations of search terms were used iteratively throughout the literature review process. Boolean operators such as AND and OR were used to refine search combinations. Only articles published in English were considered. Studies were selected according to their relevance to the mechanistic, pharmacokinetic, pathophysiological, and translational aspects of melatonin in ischemic stroke. Irrelevant publications, duplicate records, and studies unrelated to ischemic brain injury were excluded. Formal quality assessment tools were not applied due to the narrative nature of the review; however, priority was given to peer-reviewed studies published in indexed journals and to studies with significant mechanistic or translational relevance.

In addition to database searches, the reference lists of relevant original articles and review papers were manually screened to identify additional studies of interest. For pharmacological sources, including documents from agencies such as the FDA and EMA, were considered when relevant.

Special emphasis was placed on studies addressing mitochondrial regulation, oxidative stress modulation, neuroinflammatory pathways, neurovascular protection, and translational implications in ischemic stroke.

Due to the large volume of information and the continuously evolving nature of the topic, this review was designed as a narrative and thematic synthesis rather than a formal systematic review or meta-analysis. Experimental and clinical evidence were analyzed and discussed separately to improve translational interpretation.

No strict temporal restrictions were applied during literature selection, as both foundational historical studies and recent advances were considered relevant for understanding the pharmacokinetic, pathophysiological, mechanistic, and translational aspects of melatonin in ischemic stroke.

## 3. Pathophysiology of Acute Ischemic Stroke

Current understanding of ischemic stroke pathophysiology integrates evidence from both clinical studies and experimental models. While pathophysiological processes such as arterial occlusion, cerebral perfusion deficits, infarct evolution, reperfusion injury, and functional outcomes have been characterized in patients through neuroimaging and clinical studies, many of the cellular and molecular mechanisms discussed below have been derived primarily from experimental studies. Consequently, direct clinical evidence for many of the cellular pathways discussed below remains limited.

Acute ischemic stroke is defined by the American Heart Association/American Stroke Association (AHA/ASA) as “a sudden loss of neurological function caused by interruption of cerebral blood flow due to arterial occlusion, leading to ischemia and tissue damage in the affected territory” [2]. Although the etiology is multifactorial, the most frequent cause is the formation of a thrombus or embolus that obstructs a cerebral blood vessel [13].

Arterial occlusion sharply reduces cerebral blood flow (CBF). Perfusion below <17% rapidly generates an ischemic core where energy failure precipitates necrotic death within minutes [14]. Surrounding this region, the ischemic penumbra maintains partial perfusion (24–70%), preserving minimal mitochondrial function and allowing neurons to remain viable but electrically silent and metabolically suppressed [15]. Necrosis predominates in the ischemic core, defining it as a necrotic region; in contrast, apoptotic processes prevail in the surrounding ischemic penumbra, rendering it a potentially salvageable area and a therapeutic window [5,6].

**Figure 1:**
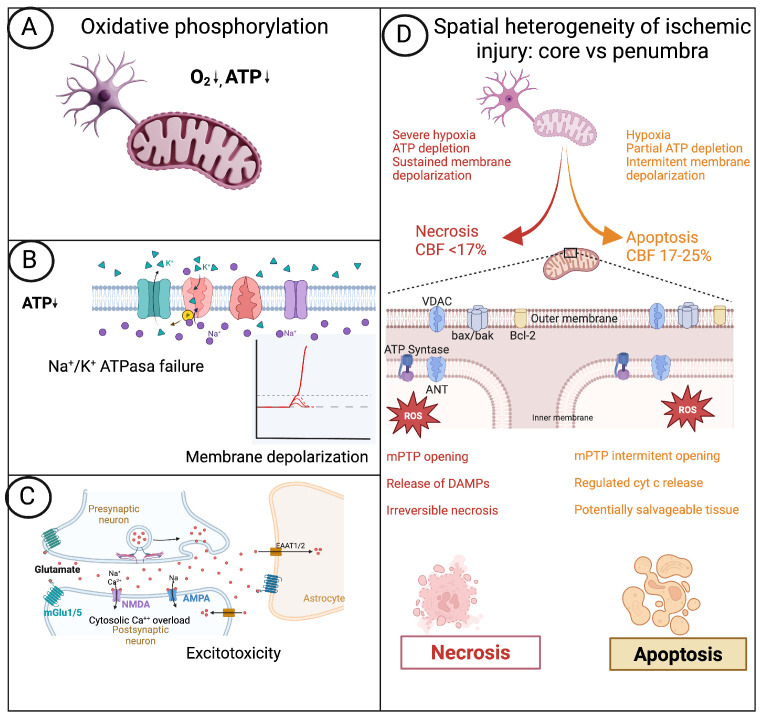
Energy failure-driven excitotoxic phenomenon leading to neuronal injury. (**A**) Interruption of cerebral blood flow reduces oxygen availability, leading to failure of oxidative phosphorylation and impaired mitochondrial ATP production. (**B**) Progressive energy depletion results in Na^+^/K^+^-ATPase failure, membrane depolarization, and ionic imbalance. (**C**) Energy failure promotes sustained glutamate release at excitotoxic synapses. (**D**) Differences in CBF and oxygen availability contribute to the distinct pathophysiological processes observed in the ischemic core and penumbra. Abbreviations: mGlu, metabotropic glutamate receptor; EAAT1/2, excitatory amino acid transporter 1/2; NMDA, *N*-methyl-D-aspartate receptor; AMPA, α-amino-3-hydroxy-5-methyl-4-isoxazolepropionic acid receptor; CBF, cerebral blood flow; Bax/Bak, Bcl-2-associated X protein/Bcl-2 homologous antagonist killer; Bcl-2, B-cell lymphoma 2; ANT, adenine nucleotide translocator; DAMPs, damage-associated molecular patterns; mPTP, mitochondrial permeability transition pore; ROS, reactive oxygen species; VDAC, voltage-dependent anion channel. Created in https://BioRender.com.

After cerebral ischemia, the hypoxic environment impairs mitochondrial oxidative phosphorylation, resulting in a marked reduction in ATP production and shifting metabolism toward cytosolic anaerobic glycolysis. This metabolic failure results in electron transport chain dysfunction [5,14,16] and triggers neuronal depolarization phenomena whose characteristics differ substantially between the ischemic core and the penumbra zones as a function of the degree of reduced cerebral blood flow (CBF), as illustrated in Figure 1 [5,16].

In the ischemic core, where CBF falls to <17% of normal, oxygen and glucose supply are virtually absent, leading to rapid and complete ATP depletion, total failure of the Na^+^/K^+^-ATPase, and sustained anoxic depolarization associated with massive calcium influx, cytotoxic edema, and necrotic cell death (Figure 1). In contrast, in the ischemic penumbra, with residual CBF of 20–40%, limited oxygen and glucose delivery persists, allowing reduced but not absent ATP levels, resulting in partial Na^+^/K^+^-ATPase dysfunction and transient, recurrent peri-infarct depolarizations [14,17].

Depolarization in both zones results in glutamate release and activation of ionotropic receptors (Figure 1), which causes an increase in intracellular calcium and the activation of calcium-dependent enzymes, resulting in oxidative stress and mitochondrial dysfunction [17,18]. NADPH oxidases are subsequently activated by multiple convergent signals, predominantly hypoxia, glutamate release, and intracellular Ca^++^ overload, thereby amplifying reactive oxygen species (ROS) production [19].

However, in the ischemic core, calcium overload triggers sustained and irreversible opening of the mitochondrial permeability transition pore (mPTP) and necrosis, whereas in the penumbra, enzyme activation and mPTP opening are partial and transient, favoring activation of pro-apoptotic signaling pathways. In this context, mitochondria act as the primary bioenergetic integrator that determines whether neuronal injury can be reversed based on the availability of oxygen and glucose ability to maintain ATP production [20,21,22].

Ischemia-induced bioenergetic and excitotoxic events result in injury that extends beyond neurons to the entire neurovascular unit (NVU).

**Figure 2:**
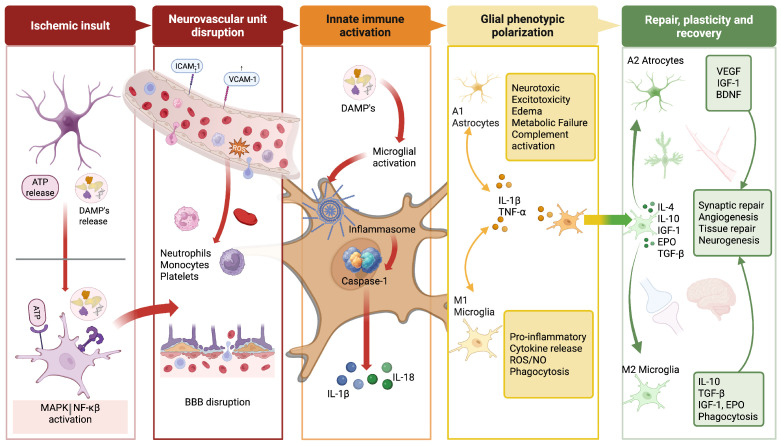
Neuroinflammatory cascade and repair mechanisms after ischemic stroke. After the initial ischemic injury, the release of DAMPs and ATP is initiated, triggering pro-inflammatory signaling pathways. Endothelial activation promotes the recruitment of inflammatory cells, BBB breakdown, and edema. Activation of the innate immune response by DAMPs and ROS promotes microglial activation, inflammasome signaling and glial polarization toward neurotoxic A1 and M1 phenotypes. A shift toward an anti-inflammatory microenvironment favors A2 and M2 phenotypes, supporting neuroprotection, plasticity, neurogenesis, and angiogenesis, thereby contributing to post-stroke recovery. Arrows indicate the direction of the pathophysiological cascade. Abbreviations: DAMPs, damage-associated molecular patterns; MAPK, mitogen-activated protein kinase; NF-κB, nuclear factor kappa B; ICAM-1, intercellular adhesion molecule 1; VCAM-1, vascular cell adhesion molecule 1; ROS, reactive oxygen species; BBB, blood–brain barrier; VEGF, vascular endothelial growth factor; IGF-1, insulin-like growth factor 1; BDNF, brain-derived neurotrophic factor; TGF-β, transforming growth factor beta; FGF, fibroblast growth factor; NRF2, nuclear factor erythroid 2-related factor 2. Created in https://BioRender.com.

Energy failure, calcium overload, and oxidative stress impair the coordinated function of neurons, astrocytes, endothelial cells, pericytes, and the extracellular matrix, promoting early BBB dysfunction. Activation of matrix metalloproteinases, along with astrocytic and microglial reactivity and endothelial tight junction disruption, increases vascular permeability, promotes vasogenic edema, and facilitates leukocyte infiltration. This neurovascular uncoupling exacerbates microcirculatory failure and amplifies secondary inflammatory injury, contributing to infarct expansion and neurological deterioration [5,23,24]. Thus, some neuroinflammatory cell signaling cascades are mainly involved in these pathophysiologic phenomena, as shown in Figure 2.

Disruptions in the NVU (Figure 2) cause early dysfunction of the BBB and endothelial activation mediated by shear-stress changes and expression of selectins, ICAM-1, VCAM-1, and chemokines that promote leukocyte recruitment [25]. Damage-associated molecular patterns (DAMPs) generated by cellular injury activate microglia, macrophages, and neutrophils, initiating innate immunity followed by adaptive responses [26].

M1-polarized microglia release pro-inflammatory mediators, including IL-1β, IL-6, TNF-α, nitric oxide (NO), reactive oxygen species (ROS), and matrix metalloproteinase-9 (MMP-9) within the first 24 h, which leads to further degradation of tight junction proteins and expansion of BBB leakage [27].

Astrocytes detect injury through pattern recognition receptors such as TLRs, as well as purinergic, complement, and glutamate receptors, triggering reactive astrogliosis. This dynamic process involves morphological, molecular, and functional remodeling and, depending on the local microenvironment, may give rise to distinct astrocytic phenotypes A1 or A2 (Figure 2). This early astrocytic response is associated with hypertrophy and increased expression of GFAP, connexin-43, and vimentin [28,29]. Under the influence of mycroglia, IL-1α, TNF-α, and C1q drive reactive astrocytes toward a neurotoxic A1 phenotype, characterized by downregulation of the glutamate transporter EAAT2 and the inwardly rectifying potassium channel Kir4.1, impairing glutamate clearance and potassium buffering; further, there are the acquisition of a deleterious secretoy profile marked by complement component C3 expression and the release of neurotoxic soluble factor that exacerbate excitotoxicity, edema, and neuronal injury [30].

Collectively, this early microglia–astrocyte crosstalk amplifies neuronal vulnerability and contributes to lesion expansion during the first hours to days following ischemic insult, setting the stage for subsequent transitions toward inflammation resolution and tissue repair in the subacute phase. Cytokines present in this environment, such as IL-1β and TNF-α, drive endothelial activation, inducing the expression of adhesion molecules such as P-selectin, E-selectin, VCAM-1, and ICAM-1 [31,32]. This process facilitates leukocyte rolling, firm adhesion, and transmigration across the vascular wall. In parallel, BBB disruption increases vascular permeability, impairing cerebral homeostasis and facilitating immune cell entry into the brain parenchyma [33,34].

Once within the parenchyma, infiltrating leukocytes release additional pro-inflammatory cytokines, metalloproteinases, and ROS, sustaining a hostile inflammatory microenvironment during ischemia. Neutrophils, in particular, amplify secondary injury through additional ROS and MMP-9 release [35]. Concurrently, platelet–leukocyte interactions promote thromboxane A_2_ production and serotonin release, as well as fibrin deposition, thereby amplifying tromboinflammation and impaired tissue perfusion [36].

DAMPs also activate NF-κB, leading to the upregulation of iNOS, COX-2, IL-1β, and IL-18, and promoting inflammasome-mediated pyroptosis [37]. In parallel, inhibition of the PI3K–Akt–mTOR pathway facilitates intrinsic apoptosis and exacerbates injury within the ischemic penumbra [38].

After 24 h, even in the absence of therapeutic reperfusion, partial resolution of hypoxia and CBF may occur due to spontaneous thrombus dissolution, recruitment of collateral circulation, compensatory vasodilatation, hemodynamic redistribution, and progressive restoration of ionic homeostasis [5,16,39]. These mechanisms contribute to the transition from injury to repair, promoting cellular reprogramming [18,40].

M2 microglia release cytokines such as IL-10 (Figure 2), as well as neurotrophic and pro-resolutive factors, including TGF-β, IGF-1, EPO, and BDNF, thereby supporting angiogenesis and tissue repair [41,42]. TGF-β activates Smad signaling, leading to upregulation of VEGF, FGF-2, and SOCS3 while concurrently inhibiting NF-κB-mediated inflammatory pathways [43]. At approximately 48 h after ischemic injury, IGF-1, FGF-2, and Nrf2 signaling pathways contribute to increased BDNF expression, supporting early neurogenesis and angiogenesis [44]. Collectively, this phenotypic shift dampens injury-associated signaling and activates resolution pathways, helping to restore local metabolic homeostasis and reprogram microglia and astrocytes toward M2 and A2 phenotypes [45,46,47].

Besides local glial and neurovascular alterations, ischemic brain injury also triggers systemic inflammatory and stress-associated neuroendocrine responses, including the release of catecholamines and growth hormone (GH), which promote metabolic adaptation and activate hypoxia and inflammation-sensitive transcription factors such as HIF-1α and NF-κB [5,18]. These pathways enhance IGF-1/IGF-1 receptor signaling in astrocytes and endothelial cells, contributing to cell survival and tissue remodeling. IGF-1 subsequently activates PI3K/Akt and ERK1/2 signaling cascades, suppressing apoptotic pathways while promoting neurogenesis and angiogenesis during the subacute and recovery phases of ischemia [48].

The convergence of neuronal activity, injury-related signals, hypoxia, and neurotrophic pathways initiates a coordinated repair and rewiring program that integrates glial reprogramming, vascular remodeling, and synaptic and structural plasticity to enable adaptive reorganization of brain networks after ischemic injury [49,50].

In the early phase, inflammatory responses inhibit axonal growth to preserve circuit stability and prevent aberrant connectivity. During the subacute phase, these systemic and transcriptional processes progressively shift toward recovery, with outcomes determined by a dynamic balance between axonal growth inhibitors and neurotrophic signaling pathways that promote neural plasticity [49,50,51].

Approximately 72 h after ischemic injury, plasticity begins with gene activation and the release of trophic factors. Neurons in the peri-infarct cortex upregulate growth-associated proteins such as GAP-43 and CAP-23, along with cytoskeletal regulators that promote axonal sprouting and synaptogenesis [49,50].

Concurrently, the expression of neurotrophic factors, including BDNF, IGF-1, and CNTF, increases. These signals activate intracellular pathways such as PI3K/Akt and MAPK/ERK in surviving neurons, promoting cell survival, growth, differentiation, and dendritic spine formation [51,52].

Recovery also involves network reorganization beyond the lesion site, including interhemispheric rebalancing and diaschisis reversal. These structural and functional changes are experience dependent, highlighting the critical role of rehabilitation in achieving meaningful functional recovery [53,54].

Concurrently, M2 microglia have been implicated in synaptic refinement through complement-dependent signaling pathways, while reparative microglial phenotypes associated with TREM2 signaling support tissue remodeling. In parallel, A2 astrocytes secrete VEGF and angiopoietins, supporting long-term angiogenesis [55,56,57]. In addition, oligodendrocyte precursor cells proliferate and contribute to the remyelination of surviving axons, thereby improving conduction efficiency within reorganized networks [58,59]. Microglia-mediated clearance of myelin creates a permissive environment for oligodendrocyte precursor cell recruitment and differentiation, promoting remyelination in the ischemic tissue [60].

At the level of the neurovascular unit, continued angiogenesis and extracellular matrix remodeling are mediated by HIF-1α, VEGF, PDGF-β, and matrix metalloproteinases, leading to gradual restoration of BBB integrity and microvascular stability [16,61,62]. Neurogenesis from the subventricular and subgranular zones contributes modestly to circuit repair and functional recovery through Wnt/β-catenin and BDNF-dependent mechanisms (Figure 2). Finally, large-scale network reorganization involves NMDA receptor-dependent synaptic plasticity and rebalancing of interhemispheric connectivity, although excessive contralesional activation may impede functional recovery [51,54].

The effectiveness of reparative and plastic processes is influenced by earlier events during ischemia, reperfusion, and secondary injury, which dynamically determine infarct evolution and tissue viability [5,16]. Beyond this phase, a sustained phase of plasticity emerges, characterized by lower intensity but persistent remodeling processes that may continue for months or even years [51].

Reperfusion may arise either as part of the natural evolution of ischemic injury, through spontaneous recanalization and collateral flow, or be rapidly induced by reperfusion therapies. Importantly, both scenarios share overlapping injury mechanisms, and effective macrovascular recanalization does not preclude persistent microvascular dysfunction, including the no-reflow phenomenon [5,16,63].

Immediately after reperfusion, reoxygenation of metabolically compromised tissue reactivates the mitochondrial respiratory chain, leading to excessive generation of reactive oxygen and nitrogen species (ROS/RNS) and impaired cellular bioenergetics, contributing to oxidative injury and metabolic disruption [34,64]. Concomitantly, Ca^2+^ overload within mitochondria promotes opening of the mitochondrial permeability transition pore (mPTP), resulting in mitochondrial swelling, dysfunction, and further ROS production [20]. This oxidative burst exacerbates lipid peroxidation, protein oxidation, and DNA damage, thereby further disrupting cellular bioenergetics and promoting neuronal vulnerability and inflammatory cell activation [19]. Collectively, these events lead to mitochondrial depolarization, ATP depletion, and activation of intrinsic apoptotic signaling cascades, ultimately contributing to delayed neuronal death within the ischemic penumbra [5,16].

Opening of the mPTP facilitates the release of cytochrome c and apoptosis-inducing factor (AIF) into the cytoplasm, triggering caspase-dependent and caspase-independent apoptotic pathways [65,66]. Mitochondrial injury, therefore, contributes to neuronal damage not only during the ischemic phase but also during reperfusion. Accordingly, reperfusion injury involves multiple forms of cell death, both regulated and unregulated, including apoptosis, necrosis, pyroptosis, and ferroptosis. Neuroimaging studies in humans have demonstrated significant infarct expansion despite technically successful recanalization, providing direct evidence that reperfusion-associated injury contributes substantially to final infarct volume and neurological outcome [67].

At the vascular level, reperfusion is accompanied by endothelial activation and dysfunction, characterized by upregulation of adhesion molecules, platelet activation, and leukocyte recruitment. During reperfusion, activated platelets act as key drivers of inflammation through multiple mechanisms, including endothelial activation and platelet adhesion, platelet–leukocyte interactions, neutrophil activation with induction of neutrophil extracellular trap (NET) formation, release of pro-inflammatory cytokines, and engagement of toll-like receptor (TLR) signaling pathways [67].

Platelet–leukocyte interactions and pericyte-mediated capillary constriction contribute to the no-reflow phenomenon, whereby microvascular perfusion remains impaired despite restoration of flow in proximal arteries [67,68]. Concurrently, inflammatory activation and leukocyte infiltration promote MMP-9 activation, which further contributes to BBB disruption, vasogenic edema, and increased risk of hemorrhagic transformation [69,70].

Ischemic stroke emerges as a highly dynamic and temporally structured process, in which early bioenergetic failure, oxidative stress, inflammation, microvascular dysfunction, delayed cell death, reperfusion injury, and subsequent phases of vascular remodeling, structural plasticity, and remyelination [5,16,49]. These mechanisms do not occur in isolation but evolve sequentially and partially overlap, creating multiple windows of vulnerability and opportunities for therapeutic modulation.

Currently, guideline-recommended therapies for acute ischemic stroke are primarily focused on restoring cerebral blood flow through reperfusion strategies, including intravenous thrombolysis and mechanical thrombectomy [2]. These interventions target the primary pathophysiological event, arterial occlusion and cerebral hypoperfusion, thereby limiting infarct progression and improving functional outcomes. In addition, antiplatelet agents, anticoagulation in selected patients, and intensive lipid-lowering therapies contribute to secondary prevention by reducing thrombotic risk, platelet activation, thromboinflammation, and vascular dysfunction. However, most of the cellular and molecular mechanisms involved in secondary ischemic injury, including mitochondrial dysfunction, oxidative stress, neuroinflammation, excitotoxicity, and programmed cell death pathways, still lack specific approved therapies.

Accordingly, effective neuroprotection is unlikely to rely on a single molecular target or a fixed time point, but rather on interventions capable of engaging distinct pathophysiological processes across the temporal continuum of ischemic injury [5,18].

Rather than acting as a cytoprotective agent for the ischemic core, melatonin should be conceptualized as a pleiotropic modulator that operates across multiple phases of ischemic stroke, ranging from early mitochondrial dysfunction and reperfusion-associated injury to delayed inflammation, apoptosis, and long-term neurovascular remodeling [10,71].

## 4. Melatonin Across the Temporal Evolution of Ischemic Stroke

**Figure 3:**
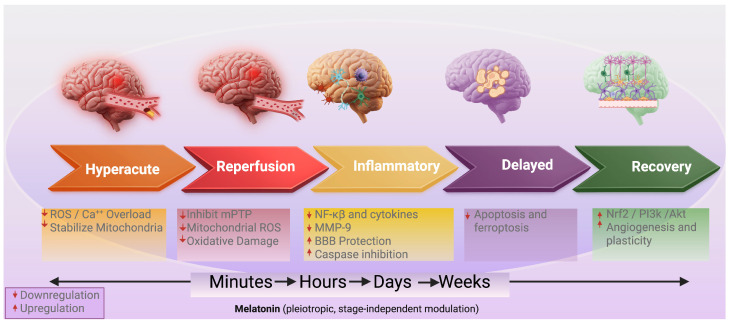
Pleiotropic actions of melatonin in ischemic stroke. Ischemic stroke evolves as a temporally structured process characterized by hyperacute mitochondrial dysfunction, reperfusion injury, inflammatory amplification, delayed cell death, and long-term neurovascular remodeling. Rather than acting directly on the ischemic core, melatonin exerts protective actions across different phases of ischemic injury. It helps preserve mitochondrial function during the hyperacute and reperfusion phases, attenuates thromboinflammatory injury within the neurovascular unit, limits apoptotic and ferroptotic cell death, and supports angiogenesis, plasticity, and neurovascular remodeling. Abbreviations: ROS, reactive oxygen species; Ca^2+^, calcium ion; mPTP, mitochondrial permeability transition pore; NF-κB, nuclear factor kappa B; MMP-9, matrix metalloproteinase 9; BBB, blood–brain barrier; Nrf2, nuclear factor erythroid 2–related factor 2; PI3K/Akt, phosphoinositide 3-kinase/protein kinase B. Created in https://BioRender.com.

During the hyperacute phase of ischemic stroke, neuronal energy failure and mitochondrial dysfunction rapidly emerge as central determinants of tissue fate. Although the necrotic core is irreversibly damaged, the surrounding penumbral tissue remains metabolically compromised yet potentially salvageable. In this early context, melatonin should not be considered a cytoprotective agent for the ischemic core but may preserve mitochondrial integrity and bioenergetic efficiency in vulnerable penumbral regions by scavenging reactive oxygen and nitrogen species, attenuating calcium overload, and stabilizing mitochondrial membranes [7,71]. The temporal organization of these early events and the potential modulatory actions of melatonin are illustrated in Figure 3.

Following recanalization, whether spontaneous or therapeutically induced, reperfusion initiates a secondary wave of injury characterized by abrupt reoxygenation, excessive mitochondrial ROS/RNS generation, calcium influx, and bioenergetic collapse associated with mitochondrial permeability transition pore opening [19,20]. In this phase, melatonin may attenuate oxidative and mitochondrial injury associated with reperfusion, thereby potentially reducing neuronal vulnerability [72,73].

As ischemia–reperfusion injury progresses into the acute and subacute inflammatory phases, endothelial activation, microglial polarization toward a pro-inflammatory phenotype, leukocyte infiltration, platelet activation, and BBB disruption collectively amplify secondary tissue damage within the neurovascular unit. Melatonin may modulate these inflammatory and thromboinflammatory processes, contributing to preservation of neurovascular integrity and microvascular perfusion [71,74].

Beyond early inflammation, delayed neuronal death in the ischemic penumbra is driven by sustained mitochondrial dysfunction and activation of intrinsic apoptotic pathways, along with emerging forms of regulated cell death, such as pyroptosis and ferroptosis [16,75,76]. In this context, experimental evidence suggests that melatonin may attenuate apoptotic and ferroptotic pathways associated with delayed ischemic injury [10,71].

In the subacute and chronic phases of ischemic injury, endogenous repair mechanisms, including angiogenesis, neurovascular remodeling, synaptic plasticity, and functional reorganization, become relevant determinants of neurological recovery [49,51]. Melatonin may support these restorative processes (Figure 3) by promoting redox homeostasis, neurovascular integrity, and adaptive plasticity, thereby contributing to long-term tissue remodeling and functional recovery [71].

Finally, evidence suggests that circadian rhythms strongly modulate both ischemic stroke pathophysiology and recovery. Reduced endogenous melatonin secretion and altered CLOCK/BMAL1 signaling contribute to circadian misalignment, which may increase oxidative stress and inflammatory dysregulation [77,78,79]. Melatonin plays a central role in circadian regulation, but its effects may extend beyond chronobiology. Experimental evidence suggests that melatonin may exert pleiotropic actions involving antioxidant, anti-inflammatory, anti-excitotoxic, and mitochondrial-protective actions, modulate apoptosis and autophagy pathways, and support neurovascular integrity and plasticity [71,72]. Through these pleiotropic mechanisms, melatonin has emerged as a possible multifunctional neuroprotective agent, although definitive clinical evidence remains limited. Overall, Figure 3 integrates these mechanisms and illustrates how melatonin may act as a pleiotropic modulator across the different temporal stages of ischemic stroke.

## 5. Mechanisms of Action of Melatonin in Ischemic Stroke

Melatonin is a hormone primarily produced by the pineal gland, which converts tryptophan into melatonin through an enzymatic pathway stimulated by norepinephrine. Melatonin is released in response to the light–dark cycle through the retinohypothalamic tract. Its secretion follows a circadian pattern, with the highest concentrations occurring between 3:00 and 4:00 a.m., reaching peak plasma levels around 200 pg/mL [80,81].

In addition to its pineal origin, extrapineal sources of melatonin, such as the retina, cerebellum, skin, gastrointestinal tract, and immune system, suggest potential local autocrine and paracrine functions [81,82].

**Figure 4:**
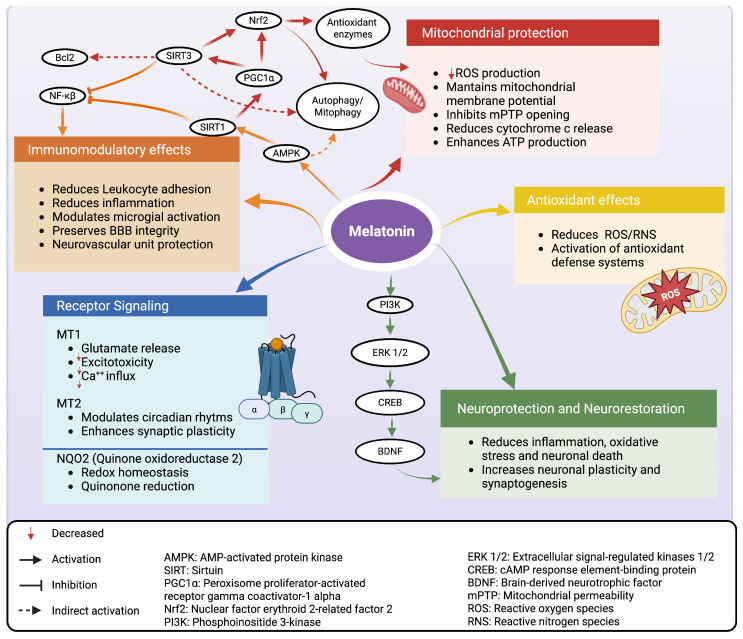
Pleiotropic mechanisms of melatonin in ischemic stroke. Experimental evidence suggests that melatonin may modulate multiple pathophysiological processes involved in ischemic brain injury, including oxidative stress, mitochondrial dysfunction, excitotoxicity, neuroinflammation, apoptosis, and neurovascular impairment. Created in https://BioRender.com.

Melatonin exerts pleiotropic molecular actions that may modulate multiple components of the ischemic cascade, potentially contributing to neuroprotective effects, as summarized in Figure 4. Melatonin exerts therapeutic effects primarily through MT1 and MT2 G protein-coupled receptors, which are distributed in the brain, retina, adipose tissue, uterus, pancreas, testicles, heart, placenta, coronary arteries, and fetal kidney [83,84]. These receptors modulate cAMP and cGMP signaling pathways, contributing to melatonin’s physiological effects [83,85].

Melatonin acts as a direct free-radical scavenger and, through receptor-dependent and independent mechanisms, reduces ROS and preserves mitochondrial integrity, thereby limiting oxidative damage. Through activation of MT1 receptors coupled to Gi proteins, melatonin modulates ion channel activity by inhibiting voltage-dependent Ca^2+^ channels and activating G protein-regulated inwardly rectifying K^+^ channels (GIRK), thereby reducing neuronal excitability and excitotoxicity [83].

Downstream signaling pathways associated with melatonin may enhance antioxidant defenses, preserve mitochondrial function, and attenuate pro-inflammatory transcription mediated by NF-κB. Melatonin has also been associated with reduced expression of pro-inflammatory cytokines, including TNF-α and IL-1β, attenuation of MMP-9 activation, and preservation of blood–brain barrier integrity [7,71].

Additionally, melatonin promotes antiapoptotic signaling through regulation of the Bcl-2 family and inhibition of pro-apoptotic pathways. Activation of PI3K/ERK ½ axis may support neuronal survival, neurovascular integrity, plasticity, and repair, collectively contributing to reduced neuronal injury, inflammation, and oxidative stress [7].

Melatonin has been reported to modulate the transcription of factors involved in anti-inflammatory, antioxidant, anti-apoptotic, and pro-survival effects, although the mechanisms involved are not fully understood (Figure 4). Melatonin modulates the cAMP/PKA pathway and downstream signaling pathways such as CREB and ERK1/2, favoring cell survival and activating the JAK2/STAT-3 and ERK/p38 pathways, increasing Bcl-2 expression and enhancing anti-apoptotic responses [84].

Via the cAMP/SIRT3/SOD2 and SIRT1/PPAR-γ pathways, melatonin increases Nrf2 expression, facilitating the synthesis of antioxidant enzymes and strengthening resistance to oxidative stress. It also inhibits NF-κB transcription, reducing inflammatory signaling [81,84,86]. JAK2, ERK, and Akt/FOXO1 activation by melatonin promote cell survival and enhance stem cell pluripotency [81,84].

Melatonin is a potent antioxidant with both direct and signaling-mediated effects. Unlike conventional antioxidants such as vitamins C and E, which generally neutralize a single reactive species, melatonin and its metabolites may scavenge multiple reactive oxygen and nitrogen species, potentially enhancing their antioxidant capacity [73,77]. Due to its amphiphilic nature, melatonin easily moves across cellular and mitochondrial membranes, enhancing mitochondrial function by increasing the activities of respiratory chain complexes I and IV and reducing electron leakage and ROS production [86]. Additionally, melatonin has been associated with protection against biochemical and endothelial injury in patients with advanced atherosclerosis [87,88].

Together, these mechanisms suggest that melatonin may modulate multiple pathophysiological processes involved in ischemic stroke, which begin within minutes and can extend for weeks or even months [89]. Experimental studies have demonstrated neuroprotective effects in several models of cerebral ischemia; however, definitive clinical efficacy remains to be established [7,8,10,26,90].

## 6. Pharmacokinetics and Translational Considerations of Melatonin

Melatonin is a small molecule (232 Da) that is rapidly absorbed and widely distributed after oral administration. It easily crosses the BBB due to its high lipophilicity and low plasma protein binding. The bioavailability of melatonin is variable, ranging from 9% to 33%, primarily due to extensive hepatic first-pass metabolism [91,92]. Endogenous melatonin distributes throughout multiple tissues, including the brain [93], with a volume of distribution (VD) of approximately 451 L [94].

Following oral administration, peak plasma concentrations are typically reached within 30 to 60 min. Melatonin is mainly metabolized in the liver by cytochrome P450 enzymes, particularly CYP1A2 and CYP2C9, into 6-hydroxymelatonin, which is subsequently conjugated with sulfate or glucuronide acid and excreted in the urine. Its plasma half-life is short, ranging from 20 to 50 min [7,95,96,97].

Despite its short plasma half-life, melatonin exerts prolonged antioxidant effects through the formation of biologically active metabolites such as N^1^-acetyl-N^2^-formyl-5-methoxykynuramine (AFMK) and N^1^-acetyl-5-methoxykynuramine (AMK), which participate in a free radical scavenging cascade and contribute to sustained intracellular antioxidant activity [77,81]. Additionally, melatonin modulates transcriptional pathways involved in oxidative stress and inflammation, resulting in biological effects that extend beyond its plasma presence [84].

Several pharmaceutical formulations are available, including sublingual tablets, sustained-release capsules, and intravenous formulations, each with distinct advantages, such as the ability to prolong half-life or enhance bioavailability. Extended-release formulations may prolong this up to 3.5–4 h, and maintain sustained plasma concentrations for approximately 6–8 h, similar to physiological nocturnal secretion [7,95,96,97].

Given the dynamic and prolonged pathophysiology of ischemic stroke, extended-release formulations and prolonged administration strategies may warrant further investigation to maintain biologically relevant melatonin concentrations across different stages of ischemic injury.

## 7. Experimental Evidence of Neuroprotective Effects

The majority of available evidence on the neuroprotective effects of melatonin is derived from experimental models, predominantly in rodents. Numerous preclinical studies have demonstrated the beneficial effects of melatonin in different models of ischemic brain injury, as summarized in Table 1.

These studies support the hypothesis that melatonin reduces infarct volume, improves neurological recovery, and exerts anti-inflammatory and antioxidant effects [7,10,26,90,118]. These benefits were observed when administered via different administration routes. Furthermore, experimental studies have demonstrated that melatonin protects the BBB, limits edema, preserves mitochondrial function, and promotes neurogenesis [7,10,81]. These findings provide a strong preclinical foundation supporting its potential for clinical translation.

## 8. Clinical Evidence and Translational Perspectives

Clinical studies evaluating melatonin in humans remain limited but increasingly suggest a potential role in modulating ischemia–reperfusion injury and related pathophysiological processes. Melatonin has demonstrated antioxidant and anti-inflammatory effects in clinical settings, along with endothelial-modulating properties, whereas anti-apoptotic mechanisms are predominantly supported by preclinical evidence [87,119,120].

Direct clinical evidence in acute ischemic stroke is mainly derived from small pilot trials (Table 2). In a randomized, double-blind, placebo-controlled study, melatonin administration (20 mg/day for 5 days) in patients with acute ischemic stroke not eligible for reperfusion therapy was associated with greater reductions in NIHSS and mRS scores at 30 and 90 days compared with placebo. However, no significant differences were observed in the proportion of patients achieving functional independence (mRS < 3). These findings suggest a potential effect on neurological recovery, although larger studies are needed to confirm clinical efficacy [121,122].

Given the limited number of clinical trials and their relatively small sizes in acute ischemic stroke, evidence from other ischemia–reperfusion settings is included to illustrate shared pathophysiological pathways relevant to cerebral injury. These data are used to support biological plausibility rather than to imply therapeutic equivalence with stroke.

Across diverse cerebral ischemia-related clinical settings (Table 3), melatonin administration has been consistently associated with reductions in oxidative stress and inflammatory markers, and in some studies with decreased tissue injury and improvements in intermediate clinical outcomes [119,123,124].

In cardiovascular settings (Table 4), melatonin has been associated with improvements in oxidative stress markers, lipid profiles, and endothelial function. In patients with metabolic syndrome and coronary artery disease, melatonin administration reduced blood pressure, LDL cholesterol, inflammatory markers, and adhesion molecules such as ICAM and VCAM, while increasing antioxidant activity and nitric oxide levels [87,129].

In acute myocardial infarction, results have been heterogeneous. Some studies report a reduction in infarct size when melatonin is administered early, whereas others do not show significant improvements in myocardial salvage index, as shown in Table 4 [119,130]. These findings suggest that the timing of administration may be an important determinant of its therapeutic efficacy, particularly in relation to ischemic onset and reperfusion status.

In surgical settings (Table 4), particularly in abdominal aortic aneurysm repair and coronary artery bypass grafting, melatonin has been associated with reduced cardiac morbidity and lower levels of reperfusion-related biomarkers such as troponin and inflammatory mediators [124,132].

Due to overlapping mechanisms involving oxidative stress, mitochondrial dysfunction, and immune dysregulation, selected clinical studies from neuroinflammatory conditions were also included, such as multiple sclerosis, in which melatonin has been associated with anti-inflammatory effects, including reductions in circulating cytokines such as TNF-α, IL-1β, and IL-6, as summarized in Table 4 [120].

Despite consistent effects on oxidative stress and inflammation, the translation of these findings into clear clinical benefits remains variable. This variability may be explained by differences in study design, patient populations, dosing regimens, and timing of administration. Differences in bioavailability, formulation, and dosing strategies may additionally contribute to variability among clinical studies [121,122].

Overall, current clinical evidence supports the safety and biological activity of melatonin; however, larger randomized controlled trials are required to establish its impact on clinical outcomes. Given the prolonged and dynamic pathophysiology of ischemic stroke, future studies should also evaluate whether sustained-release formulations may help maintain more stable melatonin concentrations and provide more durable neuroprotective and neuroreparative effects during both the acute and chronic phases of ischemic injury. The temporal evolution of stroke may require that different dosing frequencies be appropriate across the temporal stages of stroke, with more frequent administration potentially needed during the acute phase to maintain therapeutic melatonin levels, followed by lower-frequency maintenance regimens during later recovery stages. Therapeutic approaches combining intensive early administration followed by prolonged maintenance strategies may better align with the temporal progression of ischemic injury and recovery processes.

## 9. Safety, Tolerability, and Regulatory Status

Melatonin is considered a highly safe compound, with a favorable safety profile even at high doses. Both experimental and clinical studies have demonstrated excellent tolerability with minimal adverse effects [134,135].

In animal models, doses up to 100 mg/kg have not shown toxicity, and long-term administration has not caused significant organ damage or mortality [136].

Doses ranging from 1 to 20 mg/day have been used without serious side effects in human clinical trials. The most common adverse events reported are mild, including drowsiness, daytime sleepiness, headache, dizziness, and occasional gastrointestinal discomfort [136,137,138,139,140,141]. In addition, Prolonged-release melatonin has been associated with modest reductions in blood pressure, typically ranging from 3 to 6 mmHg, during nocturnal periods [142].

In humans, doses of 100 mg have been administered intravenously, without significant alterations in vital signs, laboratory parameters, or electrocardiographic findings. Estimated lethal doses extrapolated to humans are approximately 2 g intravenously and 36 g orally [134].

Nevertheless, melatonin may potentiate the sedative effects of central nervous system depressants, an aspect that should be considered in acute ischemic stroke populations, particularly in elderly patients with polypharmacy.

Melatonin has been approved in some countries for the treatment of sleep disorders, while in other regions it is primarily regulated as a dietary supplement [136,143]. This heterogeneous regulatory status may contribute to substantial variability in formulation quality, bioavailability, dosing consistency, and pharmacokinetic profiles among commercially available preparations.

Notably, the doses of melatonin administered in clinical studies are substantially lower than those commonly used in preclinical stroke models. Using the body surface area (*BSA*) conversion method proposed by [144], the human equivalent dose (*HED*) can be estimated as

$$HED\;\left(\frac{\mathrm{mg}}{\mathrm{kg}}\right)=\left(Animal\;Dose\frac{\mathrm{mg}}{\mathrm{kg}}\right)\times \left(\frac{K_{m}\;\mathrm{animal}}{K_{m}\;\mathrm{human}}\right)$$

where the *K_m_* value for rats is 6 and 37 for humans. Based on this approach, experimental doses ranging from 1 to 50 mg/kg in rats correspond to an estimated *HED* of approximately 0.162 to 8 mg/kg in humans.

Although this conversion provides a standardized interspecies scaling framework, it does not fully account for species-specific differences in pharmacokinetics, metabolism, BBB transport, or receptor sensitivity. Even under this conservative interspecies extrapolation model, the difference between doses used in preclinical and clinical studies suggests that current human trials may not achieve systemic concentrations comparable to those associated with the neuroprotective effects observed in animal models.

Most clinical studies have administered relatively low melatonin doses, commonly ranging from 5 to 25 mg, while very few studies have evaluated higher doses or intravenous administration strategies. Moreover, in the context of acute ischemic stroke, melatonin has generally been administered once daily for fewer than seven days, whereas secondary injury processes, including neuroinflammation, oxidative stress, apoptotic signaling, and glial remodeling, extend well beyond the acute phase. Both dose range and treatment duration warrant careful consideration in future trials, with particular attention to sustained administration strategies that better align with the temporal dynamics of post-ischemic brain injury, while rigorously assessing safety and tolerability.

Several clinical studies do not clearly specify the melatonin formulation used, despite the potential impact of formulation characteristics on bioavailability and pharmacokinetic variability. The short half-life of melatonin may limit the maintenance of stable melatonin concentrations throughout the prolonged pathophysiological cascade of ischemic brain injury. Future studies should systematically evaluate whether sustained-release formulations and prolonged administration strategies could provide more stable melatonin concentrations during both acute and subacute phases of stroke recovery. It is also plausible that different dosing frequencies may be required during hyperacute and chronic phases, although this remains to be established in clinical studies.

## 10. Comparison with Other Neuroprotective Treatments

Several antioxidant and neuroprotective agents, such as edaravone, *N*-acetylcysteine, and minocycline, have been investigated in acute ischemic stroke with variable and often modest clinical results. In comparison, melatonin is characterized by a favorable safety profile, low cost, wide availability, and pleiotropic biological activity [145].

Vitamin C has also been used in this clinical setting; nevertheless, melatonin demonstrates distinct pharmacological features, including high lipophilicity, efficient BBB penetration, direct and indirect antioxidant actions, and modulation of inflammatory, apoptotic, and mitochondrial pathways [81,146]. Melatonin is not limited to its role as a radical scavenger and exerts multimodal cytoprotective effects, which may be particularly relevant in the complex pathophysiology of ischemic brain injury.

Importantly, unlike several investigational neuroprotective agents that lack regulatory authorization for any clinical indication, melatonin has received approval in certain regions for sleep-related disorders. While it is not approved for acute ischemic stroke, its established clinical use in other contexts and well-documented safety profile may facilitate translational development and the design of targeted stroke trials.

## 11. Conclusions and Future Perspectives

Melatonin emerges as a biologically coherent and mechanistically versatile candidate within the evolving field of neuroprotective strategies for acute ischemic stroke. Beyond its well-established antioxidant capacity, mitochondrial preservation, modulation of neuroinflammatory signaling, regulation of apoptotic cascades, stabilization of the neurovascular unit, and regulation of immune responses should be integrated into its neuroprotective profile, leading to concurrent actions resulting in reducing post-ischemic brain injury.

Preclinical evidence consistently demonstrates reductions in infarct volume, attenuation of oxidative and inflammatory damage, and improvement in neurological recovery across experimental paradigms. Importantly, melatonin’s pharmacological profile, characterized by favorable tolerability, BBB permeability, multimodal intracellular activity, and availability in both oral and intravenous formulations, supports its potential for clinical translation. Its low cost and global accessibility further strengthen its relevance, particularly in regions where access to advanced reperfusion technologies remains limited.

Nevertheless, the transition from experimental to clinical impact requires conceptual refinement of trial design. Rather than focusing exclusively on single-dose paradigms, future investigations should address dose–response relationships, sustained administration strategies, and alignment with the temporal dynamics of ischemic injury. The persistence of neuroinflammation, mitochondrial dysfunction, glial remodeling, and BBB disruption beyond the hyperacute phase supports the need for prolonged therapeutic exposure to engage both neuroprotective and neurorestorative mechanisms.

Despite these advantages, well-designed clinical trials are required to determine the optimal dose, treatment duration, formulation, route of administration, therapeutic window, and effects on both short and long-term outcomes of melatonin in the context of acute ischemic stroke. Its use in combination with reperfusion therapies and other neuroprotective agents should also be evaluated.

In this context, the integration of mechanistic biomarkers, including IL-6, caspase-3, VEGF, and MMP-9 into clinical protocols will be essential to move beyond purely functional endpoints and establish biological target engagement. Such biomarker-guided designs may clarify whether melatonin modifies inflammatory trajectories, apoptotic signaling, and neurovascular remodeling in human stroke populations.

Regulatory recognition of melatonin’s therapeutic potential, reflected in its orphan drug designation in the European Union for non-traumatic hemorrhagic stroke (EU/3/2468), reflects its potential therapeutic role in cerebrovascular disease [146,147,148]. Although not constituting marketing authorization, this designation signals a translational pathway that may facilitate structured clinical development and rational repurposing strategies.

Future paradigms should also explore combinatorial approaches, including coadministration with reperfusion therapies or pleiotropic agents such as statins, with the aim of achieving additive or synergistic modulation of oxidative and inflammatory cascades. The profile of melatonin supports its investigation as a potential neuroprotective agent in cerebrovascular disease, such as acute ischemic stroke.

The failure of previous neuroprotective strategies in ischemic stroke reflects the complexity and temporal heterogeneity of ischemic injury. Melatonin represents one of the few candidates capable of modulating multiple interconnected pathways, mitochondrial function, redox balance, inflammatory signaling, and cell death. This integrative capacity positions melatonin as a modulator acting at the level of biological systems rather than a conventional pharmacological agent targeting a single pathway. Future studies are needed to define its efficacy. Given the temporal evolution of ischemic injury, repeated dosing strategies during the acute and subacute phases may be required to sustain therapeutic effects, although this remains to be established in clinical trials.

## Figures and Tables

**Table 1:** Preclinical Studies on the Neuroprotective Effects of Melatonin in Animal Models of Cerebral Ischemia—Indirect Evidence.

Author	Species	N/Sex	Dose (mg/kg)	Route	Dose Schedule	Main Findings
Global cerebral ischemia models						
Cho et al., 1997 [98]	Wistar rat	47/M	10	IP	0, 2, and 6 h post-ischemia	↑ Neuronal density in hippocampal CA1
Guerrero et al., 1997 [99]	Mongolian gerbil	64/M	10	IP	30 min post-surgery	↓ NO and cGMP; antioxidant effects
Joo et al., 1998 [100]	Sprague-Dawley rat	34/M	3–10	IP	1 h before and after ischemia	↓ DNA damage; ↓ infarct volume
Cuzzocrea et al., 2000 [101]	Mongolian gerbil	20/M	10	IP	30 min before and 1, 2, and 6 h post	↓ MDA; ↓ MPO; ↓ brain edema
Letechipía-Vallejo et al., 2001 [102]	Cat	18/M	10	IV	Continuous infusion for 6.5 h	↓ Neurological deficit; preservation of hippocampal neurons (CA1–CA4)
González-Burgos et al., 2007 [103]	Sprague-Dawley rat	10/M	10	IV	Continuous infusion for 6 h	Preservation of pyramidal neurons in hippocampal CA1
Letechipía-Vallejo et al., 2007 [104]	Sprague-Dawley rat	14/NR	10	IV	Continuous infusion for 6 h	>70% preservation of pyramidal neurons in CA1–CA3 and dentate hilus
García-Chávez et al., 2008 [105]	Sprague-Dawley rat	10/M	10	IV	Continuous infusion for 6 h	Preservation of prefrontal cortex cytoarchitecture
Focal cerebral ischemia models						
Kilic et al., 1999 [106]	Wistar rat	39/F	4–8	IP	30 min before and 1, 2, and 6 h post	↓ Infarct volume (~40%); improved neurological outcome
Sinha et al., 2001 [107]	Wistar rat	NR	10–40	IP	1 h before ischemia, during reperfusion, and 1 h after	↓ Infarct volume; ↓ ROS; improved neurological score
Pei et al., 2003 [108]	Sprague-Dawley rat	61/M	1.5–50	IP	30 min before ischemia	Dose-dependent reduction in infarct volume
Lee et al., 2004 [109]	Sprague-Dawley rat	32/M	5	IV	Single dose	↓ Cortical and striatal infarction; behavioral improvement
Wang et al., 2020 [110]	Rat	NR	10	IP	Post-ischemic	↓ Pyroptosis via TLR4/NF-κB pathway; enhanced neuroprotection
Liu et al., 2021 [111]	Mouse (diabetic model)	NR	10	IP	Post-ischemic administration	Improves mitochondrial function via Akt–SIRT3–SOD2 pathway; ↓ oxidative stress
Chen et al., 2022 [112]	Rat	NR	10	IP	Post-ischemic	Preserves BBB integrity via α7nAChR; ↓ HMGB1-mediated microglial activation
Hypoxic–ischemic models						
Nagai et al., 2008 [113]	Fetal rat	29/NR	4	Oral	Daily for 20 days	Mitochondrial protection under hypoxia
Kaur et al., 2008 [114]	Fetal and Wistar rat	179/NR	10	IP	Daily for 15 days	↓ VEGF and NO; hippocampal protection
Alonso-Alconada et al., 2011 [115]	Neonatal rat	NR	10	IP	Post-insult administration	↓ Apoptosis and oxidative stress
Mechanistic studies related to ischemic injury						
Reiter et al., 2000 [116]	Rat	NR	10	IP	Single dose	Potent antioxidant; ↓ structural brain damage
Andrabi et al., 2015 [117]	Mouse	NR	10	IP	Post-ischemic	Mitochondrial protection; ↓ apoptosis

IP: intraperitoneal; IV: intravenous; MCAO: middle cerebral artery occlusion; ROS: reactive oxygen species; MDA: malondialdehyde; MPO: myeloperoxidase; NO: nitric oxide; NR: not reported. ↑: increase; ↓: decrease.

**Table 2:** Clinical Studies on the Neuroprotective Effects of Melatonin in Ischemic Stroke—Direct Evidence.

Author/Year	Study Design	Patients	Melatonin Dose	Key Findings
Mehrpooya et al., 2022 [121]	Randomized, double-blind, placebo-controlled clinical trial	Adults (*N* = 65)	20 mg/day orally for 5 days	NIHSS scores showed a significantly greater reduction over 90 days in the melatonin group vs. placebo (*p* < 0.05). Lower mRS < 3 was detected between groups; however no significant differences.
Rabiee et al., 2025 [122]	Randomized, double-blind, placebo-controlled clinical trial	Adults (mean age ≈ 60 years, *N* = 70)	10 mg/day orally for 5 days	NIHSS scores were significantly reduced between day 5 and day 30 in the melatonin group (*p* = 0.001).

Abbreviations: N, sample size; NIHSS, National Institutes of Health Stroke Scale; mRS, modified Rankin Scale.

**Table 3:** Clinical studies of Melatonin in Neurological Conditions Associated with Cerebral Injury—Indirect Evidence.

Author/Year	Study Design	Patients	Melatonin Dose	Key Findings
Perinatal asphyxia				
Fulia et al., 2001 [125]	Randomized clinical trial	Term neonates (*N* = 20)	10 mg every 2 h (total 80 mg)	Mortality was lower in the melatonin group (0/10 vs. 3/10); however, statistical significance was not reported.
Ahmad et al., 2018 [126]	Randomized, double-blind clinical trial	Term or late preterm neonates (*N* = 80)	10 mg oral single dose	Mortality was significantly lower in the melatonin group (12.5% vs. 35%) (*p* = 0.03; RE 0.38, 95% CI 0.15–0.94).
Neonatal hypoxic–ischemic encephalopathy				
Aly et al., 2015 [127]	Randomized controlled trial	Term neonates (*N* = 30)	10 mg/kg/day for 5 days + hypothermia	Mortality was lower in the melatonin group (1/15 vs. 4/15), although not statistically significant (*p* = 0.33). Improved survival without neurological or developmental abnormalities at 6 months (*p* < 0.001), fewer seizures on EEG (*p* = 0.032), and reduced white matter abnormalities on MRI (*p* = 0.014).
Hemorrhagic stroke				
Dianatkhah et al., 2017 [128]	Randomized, double-blind clinical trial	Adults (mean age 57.7 ± 12.7, *N* = 40)	30 mg/day orally for 5 days	ICU stay was significantly shorter in the melatonin group (*p* = 0.041), whereas duration of mechanical ventilation showed a non-significant reduction (*p* = 0.065). Mortality was lower in the melatonin group (15% vs. 30%); statistical significance was not reported.

Abbreviations: ICU = intensive care unit; IV = intravenous; IC = intracoronary; MRI = magnetic resonance imaging; NIHSS = National Institutes of Health Stroke Scale; IL-1β = interleukin-1 beta; iNOS = inducible nitric oxide synthase; CRP = C-reactive protein; ICAM = intercellular adhesion molecule; VCAM = vascular cell adhesion molecule; RE = relative effect; CI = confidence interval. Note: Doses are reported as described in each original study. In addition to direct evidence in ischemic stroke, studies in related ischemia–reperfusion conditions are included to provide complementary translational insights.

**Table 4:** Clinical studies of melatonin evaluating melatonin in cardiovascular, metabolic, and neuroinflammatory conditions relevant to ischemic stroke—indirect evidence.

Author/Year	Study Design	Patients	Melatonin Dose	Condition/Clinical Context	Key Findings
Coronary Heart Disease					
Dwaich et al., 2016 [123]	Prospective comparative study	Adults (*N* = 45)	10–20 mg/day oral for 5 days	Acute myocardial infarction	Increased ejection fraction and decreased heart rate (*p* < 0.05). Reduced Troponin-I, IL-1β, iNOS, and caspase-3 levels
Domínguez-Rodríguez et al., 2017 [119]	Randomized double-blind placebo-controlled trial	Adults (*N* = 146)	IV 51.7 µmol pre + 8.6 µmol IC post	Acute myocardial infarction	Infarct size was significantly smaller in early-treated patients (14.6 ± 14.2 vs. 24.9 ± 9.0%; *p* = 0.003). Larger infarct size observed in late-treated patients.
Ekeloef et al., 2017 [130]	Randomized double-blind clinical trial	Adults (*N* = 48)	10 mg IC + 40 mg IV over 6 h	Acute myocardial infarction	Myocardial salvage index was similar between groups (55.3% vs. 61.5%; *p* = 0.21).
Zaslavskaia et al., 2010 [131]	Non-randomized controlled trial	Older adults (*N* = 170)	Not specified (21 days)	Cardiovascular disease (HTN + CHD)	Reduction in oxidative stress markers and blood pressure. Improved anti-ischemic and anti-anginal effects; normalization of redox balance
Javanmard et al., 2016 [87]	Randomized double-blind clinical trial	Adults (*N* = 39)	10 mg/day oral for 30 days	Coronary artery disease	Significant reductions in ICAM, VCAM, and CRP levels.
Shafiei et al., 2018 [132]	Randomized double-blind clinical trial	Adults (*N* = 88)	5 mg/day for 3 days	Coronary artery bypass grafting (CABG)	Significant reductions in Troponin I, TNF-α, lactate, and MDA (*p* ≤ 0.001). Comparable antioxidant effect to *N*-acetylcysteine.
Vascular surgery and ischemia–reperfusion injury					
Gögenur et al., 2014 [124]	Randomized double-blind clinical trial	Adults (*N* = 50)	50 mg IV intraoperative + 10 mg/day oral ×3 days	Abdominal aortic aneurysm surgery	Cardiac morbidity was significantly lower in the melatonin group (4% vs. 29%; *p* = 0.02). Troponin levels were significantly reduced (*p* = 0.036)
Zhao et al., 2018 [133]	Randomized clinical study	Adults (*N* = 60)	6 mg/day for 3 days	Carotid surgery	Reduction in inflammatory (TNF-α, IL-6) and oxidative stress markers; increased antioxidant activity.
Metabolic and vascular dysfunction					
Koziróg et al., 2011 [129]	Randomized clinical trial	Adults with metabolic syndrome (*N* = 30)	5 mg/day for 60 days	Metabolic syndrome	Significant reductions in SBP, DBP, LDL, and TBARS, with increased CAT activity (all *p* < 0.05). Improved cardiovascular risk profile.
Neuroinflammatory disordes					
Sánchez-López et al., 2018 [120]	Randomized clinical trial	Adults (*N* = 36)	25 mg/day for 90 days	Multiple sclerosis	TNF-α levels decreased significantly at 6 months (*p* < 0.05). IL-1β and IL-6 levels decreased at 3 and 6 months (*p* < 0.05).

Abbreviations: IC = intracoronary; IV = intravenous; CABG = coronary artery bypass grafting; SBP = systolic blood pressure; DBP = diastolic blood pressure; LDL = low-density lipoprotein; TBARS = thiobarbituric acid reactive substances; CAT = catalase; ICAM = intercellular adhesion molecule; VCAM = vascular cell adhesion molecule; CRP = C-reactive protein; IL = interleukin; TNF-α = tumor necrosis factor alpha; iNOS = inducible nitric oxide synthase. Note: Outcomes are reported as described in the original studies. When statistical significance was not explicitly reported, findings are presented descriptively. This table includes only clinical (human) studies; mechanistic and experimental findings are presented separately.

## Data Availability

No new data were created or analyzed in this study. Data sharing is not applicable to this article.

## Abbreviations

AMPK	AMP-activated protein kinase
AHA/ASA	American Heart Association/American Stroke Association
AIF	Apoptosis-inducing factor
Akt	Protein kinase B
AMK	N^1^-acetyl-5-methoxykynuramine
AFMK	N^1^-acetyl-N2-formyl-5-methoxykynuramine
BBB	Blood–brain barrier
BDNF	Brain-derived neurotrophic factor
CABG	Coronary artery bypass grafting
CBF	Cerebral blood flow
CI	Confidence interval
CLOCK	Circadian locomotor output cycles kaput
CNTF	Ciliary neurotrophic factor
COX-2	Cyclooxygenase-2
CRP	C-reactive protein
CREB	cAMP response element-binding protein
DAMPs	Damage-associated molecular patterns
DBP	Diastolic blood pressure
EAAT2	Excitatory amino acid transporter 2
EPO	Erythropoietin
ERK	Extracellular signal-regulated kinase
FGF-2	Fibroblast growth factor 2
GFAP	Glial fibrillary acidic protein
GH	Growth hormone
GIRK	G protein-regulated inwardly rectifying potassium channels
HED	Human equivalent dose
HIF-1α	Hypoxia-inducible factor 1-alpha
ICAM-1	Intercellular adhesion molecule 1
ICU	Intensive care unit
IGF-1	Insulin-like growth factor 1
IL	Interleukin
IL-1β	Interleukin-1 beta
IL-6	Interleukin-6
iNOS	Inducible nitric oxide synthase
IV	Intravenous
JAK2	Janus kinase 2
LDL	Low-density lipoprotein
MAPK	Mitogen-activated protein kinase
MCAO	Middle cerebral artery occlusion
MDA	Malondialdehyde
MMP-9	Matrix metalloproteinase-9
mPTP	Mitochondrial permeability transition pore
mRS	Modified Rankin Scale
NET	Neutrophil extracellular trap
NF-κB	Nuclear factor kappa B
NIHSS	National Institutes of Health Stroke Scale
NLRP3	NOD-like receptor family pyrin domain-containing 3
NO	Nitric oxide
NR	Not reported
Nrf2	Nuclear factor erythroid 2–related factor 2
NVU	Neurovascular unit
PDGF-β	Platelet-derived growth factor beta
PI3K	Phosphoinositide 3-kinase
ROS	Reactive oxygen species
RNS	Reactive nitrogen species
SBP	Systolic blood pressure
SIRT1	Sirtuin 1
SIRT3	Sirtuin 3
SOCS3	Suppressor of cytokine signaling 3
SOD2	Superoxide dismutase 2
STAT3	Signal transducer and activator of transcription 3
TBARS	Thiobarbituric acid reactive substances
TGF-β	Transforming growth factor beta
TLR	Toll-like receptor
TNF-α	Tumor necrosis factor alpha
TREM2	Triggering receptor expressed on myeloid cells 2
VEGF	Vascular endothelial growth factor
VCAM-1	Vascular cell adhesion molecule 1
VD	Volume of distribution
Wnt	Wingless-related integration site

## References

[1] Feigin V.L., Stark B.A., Johnson C.O., Roth G.A., Bisignano C., Abady G.G. (2021). Global, regional, and national burden of stroke and its risk factors, 1990–2019. Lancet Neurol..

[2] Prabhakaran S., Gonzalez N.R., Zachrison K.S., Adeoye O., Alexandrov A.W., Ansari S.A., Chapman S., Czap A.L., Dumitrascu O.M., Ishida K. (2026). 2026 Guideline for the Early Management of Patients With Acute Ischemic Stroke: A Guideline From the American Heart Association/American Stroke Association. Stroke.

[3] Neuhaus A.A., Couch Y., Hadley G., Buchan A.M. (2017). Neuroprotection in stroke: The importance of collaboration and reproducibility. Brain.

[4] O’Collins V.E., Macleod M.R., Donnan G.A., Horky L.L., van der Worp B.H., Howells D.W. (2006). 1026 Experimental treatments in acute stroke. Ann. Neurol..

[5] Dirnagl U., Iadecola C., Moskowitz M.A. (1999). Pathobiology of ischaemic stroke: An integrated view. Trends Neurosci..

[6] Iadecola C., Anrather J. (2011). Stroke research at a crossroad: Asking the brain for directions. Nat. Neurosci..

[7] Andrabi S.S., Parvez S., Tabassum H. (2015). Melatonin and Ischemic Stroke: Mechanistic Roles and Action. Adv. Pharmacol. Sci..

[8] Macleod M.R., O’Collins T., Horky L.L., Howells D.W., Donnan G.A. (2005). Systematic review and meta-analysis of the efficacy of melatonin in experimental stroke. J. Pineal Res..

[9] Shinozuka K., Staples M., Borlongan C. (2013). Melatonin-Based Therapeutics for Neuroprotection in Stroke. Int. J. Mol. Sci..

[10] Tan H.Y., Ng K.Y., Koh R.Y., Chye S.M. (2020). Pharmacological Effects of Melatonin as Neuroprotectant in Rodent Model: A Review on the Current Biological Evidence. Cell. Mol. Neurobiol..

[11] Caso J.R., Pradillo J.M., Hurtado O., Lorenzo P., Moro M.A., Lizasoain I. (2007). Toll-like receptor 4 is involved in brain damage and inflammation after experimental stroke. Circulation.

[12] (2001). Stroke Therapy Academic Industry Roundtable II (STAIR-II). Recommendations for clinical trial evaluation of acute stroke therapies. Stroke.

[13] Virmani R., Burke A.P., Kolodgie F.D., Farb A. (2002). Vulnerable plaque: The pathology of unstable coronary lesions. J. Interv. Cardiol..

[14] Lipton P. (1999). Ischemic Cell Death in Brain Neurons. Physiol. Rev..

[15] Bandera E., Botteri M., Minelli C., Sutton A., Abrams K.R., Latronico N. (2006). Cerebral blood flow threshold of ischemic penumbra and infarct core in acute ischemic stroke: A systematic review. Stroke.

[16] Lo E.H., Dalkara T., Moskowitz M.A. (2003). Mechanisms, challenges and opportunities in stroke. Nat. Rev. Neurosci..

[17] Szydlowska K., Tymianski M. (2010). Calcium, ischemia and excitotoxicity. Cell Calcium.

[18] Iadecola C., Anrather J. (2011). The immunology of stroke: From mechanisms to translation. Nat. Med..

[19] Granger D.N., Kvietys P.R. (2015). Reperfusion injury and reactive oxygen species: The evolution of a concept. Redox Biol..

[20] Baines C.P. (2009). Mitochondrial permeability transition pore. Basic Res. Cardiol..

[21] Green D.R., Reed J.C. (1998). Mitochondria and apoptosis. Science.

[22] Nicholls D.G., Budd S.L. (2000). Mitochondria and neuronal survival. Physiol. Rev..

[23] Iadecola C., Buckwalter M.S., Anrather J. (2020). Immune responses to stroke: Mechanisms, modulation, and therapeutic potential. J. Clin. Investig..

[24] Obermeier B., Daneman R., Ransohoff R.M. (2013). Development, maintenance and disruption of the blood-brain barrier. Nat. Med..

[25] Alluri H., Wilson R.L., Anasooya Shaji C., Wiggins-Dohlvik K., Patel S., Liu Y., Peng X., Beeram M.R., Davis M.L., Huang J.H. (2016). Melatonin Preserves Blood-Brain Barrier Integrity and Permeability via Matrix Metalloproteinase-9 Inhibition. PLoS ONE.

[26] Jin R., Yang G., Li G. (2010). Inflammatory mechanisms in ischemic stroke: Role of inflammatory cells. J. Leukoc. Biol..

[27] Fann D.Y., Lim Y.A., Cheng Y.L., Lok K.Z., Chunduri P., Baik S.H., Drummond G.R., Dheen S.T., Sobey C.G., Jo D.G. (2018). Evidence that NF-κB and MAPK Signaling Promotes NLRP Inflammasome Activation in Neurons Following Ischemic Stroke. Mol. Neurobiol..

[28] Escartin C., Galea E., Lakatos A., O’Callaghan J.P., Petzold G.C., Serrano-Pozo A., Steinhäuser C., Volterra A., Carmignoto G., Agarwal A. (2021). Reactive astrocyte nomenclature, definitions, and future directions. Nat. Neurosci..

[29] Sofroniew M.V. (2010). Astrocytes: Biology and pathology. Acta Neuropathol..

[30] Liddelow S.A., Barres B.A. (2017). Reactive Astrocytes: Production, Function, and Therapeutic Potential. Immunity.

[31] Przykaza Ł. (2021). Understanding the Connection Between Common Stroke Comorbidities, Their Associated Inflammation, and the Course of the Cerebral Ischemia/Reperfusion Cascade. Front. Immunol..

[32] Tirandi A., Sgura C., Carbone F., Montecucco F., Liberale L. (2023). Inflammatory biomarkers of ischemic stroke. Intern. Emerg. Med..

[33] Tobin M.K., Bonds J.A., Minshall R.D., Pelligrino D.A., Testai F.D., Lazarov O. (2014). Neurogenesis and inflammation after ischemic stroke: What is known and where we go from here. J. Cereb. Blood Flow Metab..

[34] Zhang M., Liu Q., Meng H., Duan H., Liu X., Wu J., Gao F., Wang S., Tan R., Yuan J. (2024). Ischemia-reperfusion injury: Molecular mechanisms and therapeutic targets. Signal Transduct. Target Ther..

[35] Jickling G.C., Liu D., Ander B.P., Stamova B., Zhan X., Sharp F.R. (2015). Targeting neutrophils in ischemic stroke: Translational insights from experimental studies. J. Cereb. Blood Flow Metab..

[36] del Zoppo G.J. (2009). Inflammation and the neurovascular unit in the setting of focal cerebral ischemia. Neuroscience.

[37] Anderson C.M., Swanson R.A. (2000). Astrocyte glutamate transport: Review of properties, regulation, and physiological functions. Glia.

[38] Zhao Y., Gan Y., Xu G., Liu D. (2020). MSCs-Derived Exosomes Attenuate Acute Brain Injury and Inhibit Microglial Inflammation by Reversing CysLT2R-ERK1/2 Mediated Microglia M1 Polarization. Neurochem. Res..

[39] Liebeskind D.S. (2003). Collateral circulation. Stroke.

[40] Carmichael S.T. (2012). Brain excitability in stroke: The yin and yang of stroke progression. Arch. Neurol..

[41] Haupt M., Gerner S.T., Doeppner T.R. (2024). The dual role of microglia in ischemic stroke and its modulation via extracellular vesicles and stem cells. Neuroprotection.

[42] Lyu J., Xie D., Bhatia T.N., Leak R.K., Hu X., Jiang X. (2021). Microglial/Macrophage polarization and function in brain injury and repair after stroke. CNS Neurosci. Ther..

[43] Seo J.H., Yu J.H., Suh H., Kim M.S., Cho S.R. (2013). Fibroblast growth factor-2 induced by enriched environment enhances angiogenesis and motor function in chronic hypoxic-ischemic brain injury. PLoS ONE.

[44] Chen J., Zhang C., Jiang H., Li Y., Zhang L., Robin A., Katakowski M., Lu M., Chopp M. (2005). Atorvastatin induction of VEGF and BDNF promotes brain plasticity after stroke in mice. J. Cereb. Blood Flow Metab..

[45] Hu X., Leak R.K., Shi Y., Suenaga J., Gao Y., Zheng P., Chen J. (2015). Microglial and macrophage polarization—New prospects for brain repair. Nat. Rev. Neurol..

[46] Pekny M., Pekna M., Messing A., Steinhäuser C., Lee J.M., Parpura V., Hol E.M., Sofroniew M.V., Verkhratsky A. (2016). Astrocytes: A central element in neurological diseases. Acta Neuropathol..

[47] Zhu H., Hu S., Li Y., Sun Y., Xiong X., Hu X., Chen J., Qiu S. (2022). Interleukins and Ischemic Stroke. Front. Immunol..

[48] Liu T., Li X., Zhou X., Chen W., Wen A., Liu M., Ding Y. (2024). PI3K/AKT signaling and neuroprotection in ischemic stroke: Molecular mechanisms and therapeutic perspectives. Neural Regen. Res..

[49] Carmichael S.T. (2006). Cellular and molecular mechanisms of neural repair after stroke: Making waves. Ann. Neurol..

[50] Benowitz L.I., Carmichael S.T. (2010). Promoting axonal rewiring to improve outcome after stroke. Neurobiol. Dis..

[51] Murphy T.H., Corbett D. (2009). Plasticity during stroke recovery: From synapse to behaviour. Nat. Rev. Neurosci..

[52] Huang E.J., Reichardt L.F. (2001). Neurotrophins: Roles in Neuronal Development and Function. Annu. Rev. Neurosci..

[53] Holtmaat A., Svoboda K. (2009). Experience-dependent structural synaptic plasticity in the mammalian brain. Nat. Rev. Neurosci..

[54] Krakauer J.W., Carmichael S.T., Corbett D., Wittenberg G.F. (2012). Getting neurorehabilitation right: What can be learned from animal models?. Neurorehabil. Neural Repair.

[55] Liddelow S.A., Guttenplan K.A., Clarke L.E., Bennett F.C., Bohlen C.J., Schirmer L., Bennett M.L., Münch A.E., Chung W.S., Peterson T.C. (2017). Neurotoxic reactive astrocytes are induced by activated microglia. Nature.

[56] Schafer D.P., Lehrman E.K., Kautzman A.G., Koyama R., Mardinly A.R., Yamasaki R., Ransohoff R.M., Greenberg M.E., Barres B.A., Stevens B. (2012). Microglia Sculpt Postnatal Neural Circuits in an Activity and Complement-Dependent Manner. Neuron.

[57] Stevens B., Allen N.J., Vazquez L.E., Howell G.R., Christopherson K.S., Nouri N., Micheva K.D., Mehalow A.K., Huberman A.D., Stafford B. (2007). The Classical Complement Cascade Mediates CNS Synapse Elimination. Cell.

[58] Garcia-Martin G., Alcover-Sanchez B., Wandosell F., Cubelos B. (2022). Pathways Involved in Remyelination after Cerebral Ischemia. Curr. Neuropharmacol..

[59] Huang S., Ren C., Luo Y., Ding Y., Ji X., Li S. (2023). New insights into the roles of oligodendrocytes regulation in ischemic stroke recovery. Neurobiol. Dis..

[60] Miron V.E., Boyd A., Zhao J.W., Yuen T.J., Ruckh J.M., Shadrach J.L. (2013). Microglia in remyelination. Nat. Neurosci..

[61] Rosenberg G.A. (2009). Matrix metalloproteinases. Lancet Neurol..

[62] Armulik A., Genové G., Betsholtz C. (2011). Pericytes: Developmental, Physiological, and Pathological Perspectives, Problems, and Promises. Dev. Cell.

[63] Jia M., Jin F., Li S., Ren C., Ruchi M., Ding Y. (2024). No-reflow phenomenon. CNS Neurosci. Ther..

[64] Fang S., Huang W., Qu X., Chai W. (2024). Mitochondria in ischemia. Front. Neurosci..

[65] Yang J.L., Mukda S., Chen S.D. (2018). Mitochondria in stroke. Redox Biol..

[66] Saini S.K., Singh D. (2024). Mitochondrial mechanisms. Mitochondrion.

[67] Stoll G., Nieswandt B., Schuhmann M.K. (2024). Thrombo-inflammatory mechanisms. Neurol. Res. Pract..

[68] Schuhmann M.K., Stoll G., Bieber M., Vögtle T., Hofmann S., Klaus V. (2020). Platelet activity in stroke. Circ. Res..

[69] Rosenberg G.A., Estrada E.Y., Dencoff J.E. (1998). Matrix metalloproteinases and TIMPs are associated with blood-brain barrier opening after reperfusion in rat brain. Stroke.

[70] Cunningham L.A., Wetzel M., Rosenberg G.A. (2005). Multiple roles for MMPs and TIMPs in cerebral ischemia. Glia.

[71] Reiter R.J., Sharma R., Rosales-Corral S.A., Coto-Montes A., Boga J.A., Vriend J. (2020). Advances in Characterizing Recently-Identified Molecular Actions of Melatonin: Clinical Implications. Approaching Complex Diseases.

[72] Reiter R.J., Tan D.X., Manchester L.C., El-Sawi M.R. (2002). Melatonin reduces oxidant damage and promotes mitochondrial respiration: Implications for aging. Ann. N. Y. Acad. Sci..

[73] Leon J., Acuña-Castroviejo D., Sainz R.M., Mayo J.C., Tan D.X., Reiter R.J. (2004). Melatonin and mitochondrial function. Life Sci..

[74] Carrillo-Vico A., Reiter R.J., Lardone P.J., Herrera J.L., Fernández-Montesinos R., Guerrero J.M., Pozo D. (2006). The modulatory role of melatonin on immune responsiveness. Curr. Opin. Investig. Drugs.

[75] Stockwell B.R., Friedmann Angeli J.P., Bayir H., Bush A.I., Conrad M., Dixon S.J., Fulda S., Gascón S., Hatzios S.K., Kagan V.E. (2017). Ferroptosis: A Regulated Cell Death Nexus Linking Metabolism, Redox Biology, and Disease. Cell.

[76] Galluzzi L., Vitale I., Aaronson S.A., Abrams J.M., Adam D., Agostinis P., Alnemri E.S., Altucci L., Amelio I., Andrews D.W. (2018). Molecular mechanisms of cell death: Recommendations of the Nomenclature Committee on Cell Death 2018. Cell Death Differ..

[77] Tan D.X., Manchester L.C., Esteban-Zubero E., Zhou Z., Reiter R.J. (2015). Melatonin as a Potent and Inducible Endogenous Antioxidant: Synthesis and Metabolism. Molecules.

[78] Musiek E.S., Holtzman D.M. (2016). Mechanisms linking circadian clocks, sleep, and neurodegeneration. Science.

[79] Iadecola C. (2017). The Neurovascular Unit Coming of Age: A Journey through Neurovascular Coupling in Health and Disease. Neuron.

[80] Guerrero J.M., Carrillo-Vico A., Lardone P.J. (2007). La Melatonina. Investig. Cienc..

[81] Reiter R.J., Tan D.X., Galano A. (2014). Melatonin: Exceeding expectations. Physiology.

[82] Tan D.X., Hardeland R., Manchester L.C., Korkmaz A., Ma S., Rosales-Corral S., Reiter R.J. (2012). Functional roles of melatonin in plants, and perspectives in nutritional and agricultural science. J. Exp. Bot..

[83] Dubocovich M.L., Delagrange P., Krause D.N., Sugden D., Cardinali D.P., Olcese J. (2010). International Union of Basic and Clinical Pharmacology. LXXV. Nomenclature, classification, and pharmacology of G protein-coupled melatonin receptors. Pharmacol. Rev..

[84] Cecon E., Oishi A., Jockers R. (2018). Melatonin receptors: Molecular pharmacology and signalling in the context of system bias: Melatonin receptor system bias. Br. J. Pharmacol..

[85] Legros C., Devavry S., Caignard S., Tessier C., Delagrange P., Ouvry C., Boutin J.A., Nosjean O. (2014). Melatonin MT1 and MT2 receptors display different molecular pharmacologies only in the G-protein coupled state. Br. J. Pharmacol..

[86] Chattree V., Singh K., Singh K., Goel A., Maity A., Lone A. (2022). A comprehensive review on modulation of SIRT1 signaling pathways in the immune system of COVID-19 patients by phytotherapeutic melatonin and epigallocatechin-3-gallate. J. Food Biochem..

[87] Javanmard S.H., Heshmat-Ghahdarijani K., Mirmohammad-Sadeghi M., Sonbolestan S.A., Ziayi A. (2016). The effect of melatonin on endothelial dysfunction in patient undergoing coronary artery bypass grafting surgery. Adv. Biomed. Res..

[88] Cho J.H., Bhutani S., Kim C.H., Irwin M.R. (2021). Anti-inflammatory effects of melatonin: A systematic review and meta-analysis of clinical trials. Brain Behav. Immun..

[89] Barone F.C., Feuerstein G.Z. (1999). Inflammatory mediators and stroke: New opportunities for novel therapeutics. J. Cereb. Blood Flow Metab..

[90] Ramos E., Patiño P., Reiter R.J., Gil-Martín E., Marco-Contelles J., Parada E., de Los Rios C., Romero A., Egea J. (2017). Ischemic brain injury: New insights on the protective role of melatonin. Free Radic. Biol. Med..

[91] Di W.L., Kadva A., Johnston A., Silman R.N. (1997). Variable Bioavailability of Oral Melatonin. Engl. J. Med..

[92] Harpsøe N.G., Andersen L.P.H., Gögenur I., Rosenberg J. (2015). Clinical pharmacokinetics of melatonin: A systematic review. Eur. J. Clin. Pharmacol..

[93] Kopin I.J., Pare C.M., Axelrod J., Weissbach H. (1961). The fate of melatonin in animals. J. Biol. Chem..

[94] López-Gamboa M., Canales-Gómez J.S., Castro-Sandoval T. (2010). Bioavailability of Long Acting Capsules of Melatonin in Mexican Healthy Volunteers. J. Biotechnol. Biomater..

[95] European Medicines Agency (EMA) (2007). Assessment Report for Circadin (Melatonin).

[96] Andersen L.P.H., Werner M.U., Rosenkilde M.M., Harpsøe N.G., Fuglsang H., Rosenberg J., Gögenur I. (2016). Pharmacokinetics of oral and intravenous melatonin in healthy volunteers. BMC Pharmacol. Toxicol..

[97] Fourtillan J.B., Brisson A.M., Gobin P., Ingrand I., Decourt J.P., Girault J. (2000). Bioavailability of melatonin in humans after day-time administration of D(7) melatonin. Biopharm. Drug Dispos..

[98] Cho S., Joh T.H., Baik H.H., Dibinis C., Volpe B.T. (1997). Melatonin administration protects CA1 hippocampal neurons after transient forebrain ischemia in rats. Brain Res..

[99] Guerrero J.M., Reiter R.J., Ortiz G.G., Pablos M.I., Sewerynek E., Chuang J.I. (1997). Melatonin prevents increases in neural nitric oxide and cyclic GMP production after transient brain ischemia and reperfusion in the Mongolian gerbil (Meriones unguiculatus). J. Pineal Res..

[100] Joo J.Y., Uz T., Manev H. (1998). Opposite effects of pinealectomy and melatonin administration on brain damage following cerebral focal ischemia in rat. Restor. Neurol. Neurosci..

[101] Cuzzocrea S., Costantino G., Gitto E., Mazzon E., Fulia F., Serraino I., Cordaro S., Barberi I., De Sarro A., Caputi A.P. (2000). Protective effects of melatonin in ischemic brain injury. J. Pineal Res..

[102] Letechipía-Vallejo G., González-Burgos I., Cervantes M. (2001). Neuroprotective effect of melatonin on brain damage induced by acute global cerebral ischemia in cats. Arch. Med. Res..

[103] González-Burgos I., Letechipía-Vallejo G., López-Loeza E., Moralí G., Cervantes M. (2007). Long-term study of dendritic spines from hippocampal CA1 pyramidal cells, after neuroprotective melatonin treatment following global cerebral ischemia in rats. Neurosci. Lett..

[104] Letechipía-Vallejo G., López-Loeza E., Espinoza-González V., González-Burgos I., Olvera-Cortés M.E., Moralí G., Cervantes M. (2007). Long-term morphological and functional evaluation of the neuroprotective effects of post-ischemic treatment with melatonin in rats. J. Pineal Res..

[105] García-Chávez D., González-Burgos I., Letechipía-Vallejo G., López-Loeza E., Moralí G., Cervantes M. (2008). Long-term evaluation of cytoarchitectonic characteristics of prefrontal cortex pyramidal neurons, following global cerebral ischemia and neuroprotective melatonin treatment, in rats. Neurosci. Lett..

[106] Kilic E., Ozdemir Y.G., Bolay H., Keleştimur H., Dalkara T. (1999). Pinealectomy aggravates and melatonin administration attenuates brain damage in focal ischemia. J. Cereb. Blood Flow Metab..

[107] Sinha K., Degaonkar M.N., Jagannathan N.R., Gupta Y.K. (2001). Effect of melatonin on ischemia reperfusion injury induced by middle cerebral artery occlusion in rats. Eur. J. Pharmacol..

[108] Pei Z., Pang S.F., Cheung R.T.F. (2003). Pretreatment with melatonin reduces volume of cerebral infarction in a rat middle cerebral artery occlusion stroke model. J. Pineal Res..

[109] Lee E.J., Wu T.S., Lee M.Y., Chen T.Y., Tsai Y.Y., Chuang J.I., Chang G.L., Hsu H.Y., Lin Y.S. (2004). Delayed treatment with melatonin enhances electrophysiological recovery following transient focal cerebral ischemia in rats. J. Pineal Res..

[110] Wang K., Ru J., Zhang H., Chen J., Lin X., Lin Z., Wen C., Wu X., Lin B., Huang L. (2020). Melatonin enhances the therapeutic effect of plasma exosomes against cerebral ischemia-induced pyroptosis through the TLR4/NF-κB pathway. Front. Neurosci..

[111] Liu L., Cao Q., Gao W., Li B., Xia Z., Zhao B. (2021). Melatonin protects against focal cerebral ischemia-reperfusion injury in diabetic mice by ameliorating mitochondrial impairments: Involvement of the Akt-SIRT3-SOD2 signaling pathway. Aging.

[112] Chen S., Sun Y., Li F., Zhang X., Hu X., Zhao X., Zhou Y., Wang J., Liu Y. (2022). Modulation of α7nAChR by melatonin alleviates ischemia- and reperfusion-compromised integrity of the blood–brain barrier through inhibiting HMGB1-mediated microglia activation and CRTC1-mediated neuronal loss. Cell. Mol. Neurobiol..

[113] Nagai R., Watanabe K., Wakatsuki A., Hamada F., Shinohara K., Hayashi Y., Imamura R., Fukaya T. (2008). Melatonin preserves fetal growth in rats by protecting against ischemia/reperfusion-induced oxidative/nitrosative mitochondrial damage in the placenta. J. Pineal Res..

[114] Kaur C., Sivakumar V., Lu J., Tang F.R., Ling E.A. (2008). Melatonin attenuates hypoxia-induced ultrastructural changes and increased vascular permeability in the developing hippocampus. Brain Pathol..

[115] Alonso-Alconada D., Alvarez A., Lacalle J., Hilario E. (2012). Histological study of the protective effect of melatonin on neural cells after neonatal hypoxia-ischemia. Histol. Histopathol..

[116] Reiter R.J., Tan D.X., Osuna C., Gitto E. (2000). Actions of melatonin in the reduction of oxidative stress: A review. J. Biomed. Sci..

[117] Andrabi S.A., Sayeed I., Siemen D., Wolf G., Horn T.F.W. (2004). Direct inhibition of the mitochondrial permeability transition pore: A possible mechanism responsible for anti-apoptotic effects of melatonin. FASEB J..

[118] Ramos E., Farré-Alins V., Egea J., López-Muñoz F., Reiter R.J., Romero A. (2020). Melatonin’s efficacy in stroke patients; a matter of dose? A systematic review. Toxicol. Appl. Pharmacol..

[119] Domínguez-Rodríguez A., Abreu-González P., de la Torre-Hernandez J.M., Consuegra-Sanchez L., Piccolo R., Gonzalez-Gonzalez J., Garcia-Camarero T., Garcia-Saiz M.D.M., Aldea-Perona A., Reiter R.J. (2017). Usefulness of Early Treatment With Melatonin to Reduce Infarct Size in Patients With ST-Segment Elevation Myocardial Infarction Receiving Percutaneous Coronary Intervention (From the Melatonin Adjunct in the Acute Myocardial Infarction Treated With Angioplasty Trial. Am. J. Cardiol..

[120] Sánchez-López A.L., Ortiz G.G., Pacheco-Moises F.P., Mireles-Ramírez M.A., Bitzer-Quintero O.K., Delgado-Lara D.L.C., Ramírez-Jirano L.J., Velázquez-Brizuela I.E. (2018). Efficacy of Melatonin on Serum Pro-inflammatory Cytokines and Oxidative Stress Markers in Relapsing Remitting Multiple Sclerosis. Arch. Med. Res..

[121] Mehrpooya M., Mazdeh M., Rahmani E., Khazaie M., Ahmadimoghaddam D. (2022). Melatonin supplementation may benefit patients with acute ischemic stroke not eligible for reperfusion therapies: Results of a pilot study. J. Clin. Neurosci..

[122] Rabiee M., Abdolhoseinpour H., Mojtahedzadeh A., Mojtahedzadeh M., Shiemorteza M., Ghanbarzamani F. (2025). Clinical investigation of protective effects of melatonin on patients with acute ischemic stroke. Arch. Anesth. Crit. Care.

[123] Dwaich K.H., Al-Amran F.G.Y., Al-Sheibani B.I.M., Al-Aubaidy H.A. (2016). Melatonin effects on myocardial ischemia–reperfusion injury: Impact on the outcome in patients undergoing coronary artery bypass grafting surgery. Int. J. Cardiol..

[124] Gögenur I., Kücükakin B., Panduro Jensen L., Reiter R.J., Rosenberg J. (2014). Melatonin reduces cardiac morbidity and markers of myocardial ischemia after elective abdominal aortic aneurism repair: A randomized, placebo-controlled, clinical trial. J. Pineal Res..

[125] Fulia F., Gitto E., Cuzzocrea S., Reiter R.J., Dugo L., Gitto P., Barberi S., Cordaro S., Barberi I. (2001). Increased levels of malondialdehyde and nitrite/nitrate in the blood of asphyxiated newborns: Reduction by melatonin. J. Pineal Res..

[126] Ahmad Q.M., Chishti A.L., Waseem N. (2018). Role of melatonin in management of hypoxic ischaemic encephalopathy in newborns: A randomized controlled trial. J. Pak. Med. Assoc..

[127] Aly H., Elmahdy H., El-Dib M., Rowisha M., Awny M., El-Gohary T., El-Mashad A.R., Hamisa M., El-Saied M. (2015). Melatonin use for neuroprotection in perinatal asphyxia: A randomized controlled pilot study. J. Perinatol..

[128] Dianatkhah M., Najafi A., Sharifzadeh M., Ahmadi A., Sharifnia H., Mojtahedzadeh M., Hosseini M., Abdollahi M. (2017). Melatonin supplementation may improve the outcome of patients with hemorrhagic stroke in the intensive care unit. J. Res. Pharm. Pract..

[129] Koziróg M., Poliwczak A.R., Duchnowicz P., Koter-Michalak M., Sikora J., Broncel M. (2011). Melatonin treatment improves blood pressure, lipid profile, and parameters of oxidative stress in patients with metabolic syndrome. J. Pineal Res..

[130] Ekeloef S., Halladin N., Fonnes S., Jensen S.E., Zaremba T., Rosenberg J., Jonsson G., Aarøe J., Gasbjerg L.S., Rosenkilde M.M. (2017). Effect of Intracoronary and Intravenous Melatonin on Myocardial Salvage Index in Patients with ST-Elevation Myocardial Infarction: A Randomized Placebo Controlled Trial. J. Cardiovasc. Transl. Res..

[131] Zaslavskaia R.M., Shcherban’ E.A., Lilitsa G.V., Logvinenko S.I. (2010). Melatonin in the combined treatment of cardiovascular diseases. Klin. Med..

[132] Shafiei E., Bahtoei M., Raj P., Ostovar A., Iranpour D., Akbarzadeh S., Shahryari H., Anvaripour A., Tahmasebi R., Netticadan T. (2018). Effects of N-acetyl cysteine and melatonin on early reperfusion injury in patients undergoing coronary artery bypass grafting: A randomized, open-labeled, placebo-controlled trial. Medicine.

[133] Zhao Z., Lu C., Li T., Wang W., Ye W., Zeng R., Ni L., Lai Z., Wang X., Liu C. (2018). The protective effect of melatonin on brain ischemia and reperfusion in rats and humans: In vivo assessment and a randomized controlled trial. J. Pineal Res..

[134] Andersen L.P., Werner M.U., Rosenkilde M.M., Fenger A.Q., Petersen M.C., Rosenberg J., Gögenur I. (2016). Pharmacokinetics of high-dose intravenous melatonin in humans. J. Clin. Pharmacol..

[135] Wade A.G., Ford I., Crawford G., McConnachie A., Nir T., Laudon M. (2010). Melatonin and insomnia. BMC Med..

[136] European Medicines Agency (EMA) (2018). Assessment Report: Slenyto (Melatonin).

[137] Wade A.G., Crawford G., Ford I., McConnachie A., Nir T., Laudon M., Zisapel N. (2011). Prolonged release melatonin in the treatment of primary insomnia: Evaluation of the age cut-off for short- and long-term response. Curr. Med. Res. Opin..

[138] Malow B.A., Findling R.L., Schroder C.M., Maras A., Breddy J., Nir T., Zisapel N., Gringras P. (2021). Sleep, Growth, and Puberty After 2 Years of Prolonged-Release Melatonin in Children With Autism Spectrum Disorder. J. Am. Acad. Child Adolesc. Psychiatry.

[139] Gringras P., Nir T., Breddy J., Frydman-Marom A., Findling R.L. (2017). Efficacy and Safety of Pediatric Prolonged-Release Melatonin for Insomnia in Children With Autism Spectrum Disorder. J. Am. Acad. Child Adolesc. Psychiatry.

[140] Buscemi N., Vandermeer B., Hooton N., Pandya R., Tjosvold L., Hartling L. (2006). Efficacy and safety of exogenous melatonin for secondary sleep disorders and sleep disorders accompanying sleep restriction: Meta-analysis. BMJ.

[141] Gringras P., Findling R.L. Efficacy and Safety of Circadin^®^ in the Treatment of Sleep Disturbances in Children with Neurodevelopment Disabilities; ClinicalTrials.gov Identifier: NCT01906866. NCT01906866.

[142] Lemoine P., Zisapel N. (2012). Prolonged-release formulation of melatonin (Circadin) for the treatment of insomnia. Expert Opin. Pharmacother..

[143] Grossman E., Laudon M., Zisapel N. (2011). Effect of melatonin on nocturnal blood pressure: Meta-analysis of randomized controlled trials. Vasc. Health Risk Manag..

[144] Nair A.B., Jacob S. (2016). A simple practice guide for dose conversion between animals and human. J. Basic Clin. Pharm..

[145] Sánchez-Barceló E.J., Rueda N., Mediavilla M.D., Martinez-Cue C., Reiter R.J. (2017). Clinical Uses of Melatonin in Neurological Diseases and Mental and Behavioural Disorders. Curr. Med. Chem..

[146] Paredes S.D., Korkmaz A., Manchester L.C., Tan D.X., Reiter R.J. (2009). Phytomelatonin: A review. J. Exp. Bot..

[147] Wu H.J., Wu C., Niu H.J., Wang K., Mo L.J., Shao A.W. (2017). Neuroprotective Mechanisms of Melatonin in Hemorrhagic Stroke. Cell. Mol. Neurobiol..

[148] European Medicines Agency (EMA) EU/3/21/2468—Orphan Designation for Treatment of Non-Traumatic Spontaneous Intracerebral Haemorrhage (Melatonin). https://www.ema.europa.eu/en/medicines/human/orphan-designations/eu-3-21-2468#overview.

